# A case report on appendicular diverticulitis: Eluding traditional diagnosis

**DOI:** 10.1016/j.ijscr.2024.109563

**Published:** 2024-03-19

**Authors:** Midhun Mathew, Mohammed Fajar Al Sadiq, Vinod Gopalkrishna Pillai

**Affiliations:** a Department of General Surgery, Beaumont Hospital, Beaumont Road Dublin 9, Ireland; b Department of General Surgery, Believers Church Medical College Hospital, Thiruvalla, Kerala 689103, India

**Keywords:** Acute diverticulitis, Acute appendicitis, Appendectomy, Histopathology, Case report, Right iliac fossa pain

## Abstract

**Introduction and importance:**

Diverticula of the vermiform appendix are rare entities. Although the pathogenesis and natural course of appendiceal diverticulitis (AD) are different from acute appendicitis, AD is treated like acute appendicitis because of similar clinical manifestations and low incidence.

**Case presentation:**

We describe cases of two male patients of different ages who respectively underwent elective laparoscopic appendectomy and an emergent laparoscopic appendectomy in a multi-speciality hospital in Kerala, India. Both of them had acute appendicitis, as confirmed by imaging and laboratory testing. Subsequent histopathological examination revealed AD.

**Clinical discussion:**

AD is an uncommon but potentially more serious form of appendiceal disease that can mimic acute appendicitis or malignancy. The first patient mimicked a malignancy with the mass presentation, while the second case presented like appendicitis. Unlike colonic diverticula, AD diverticula are typically not detectable by imaging or colonoscopy, which poses a diagnostic challenge.

**Conclusion:**

In patients who present with lower abdominal pain or who may have appendicitis, AD should be considered as a differential diagnosis.

## Highlights

• Appendicular diverticulitis (AD) is a rare but significant form of appendiceal disease that can mimic acute appendicitis.
• AD is a distinct disease process that presents differently from classic appendicitis in terms of age, symptom onset, surgical findings, and histopathological analysis.
• Symptomatic AD is treated with appendectomy.
• Subsequent histopathological examination reveals a diagnosis of AD.
• AD is typically documented in case reports.

## Abbreviations

AD: Appendiceal Diverticulitis
CT: Computed Tomography
RIF: Right Iliac Fossa
OPD: Outpatients Department
OGD: Oesophago-Gastro-Duodenoscopy
DM: Diabetes Mellitus

## 1 Introduction

Appendiceal diverticulitis (AD) was initially described by pathologist Kelynack in 1893 [1]. Diverticula are blind-ended protrusions that connect to the bowel lumen through muscular mural defects. They can also arise from the vermiform appendix. This anatomical disruption in the appendiceal wall is comparable to that observed in colon diverticula. True diverticula contains outpouching of all the layers of the bowel wall, while pseudodiverticula are mucosal outpouchings. AD is thought to arise secondary to orifice obstruction, with subsequent inflammation leading to pseudodiverticula and perforation [2]. AD has been deemed extremely uncommon since its incidence, ranging from 0.004 % to 2.1 % [3]. The number of layers that herniate through the appendix wall determines its classification as acquired or congenital. Due to their extreme rarity and involvement of all layers of the wall, congenital diverticula are typically found at the anti-mesenteric edge and are linked to a lower perforation rate. In contrast, acquired diverticula are typically found in the distal third of the appendix (60 %) on the mesenteric edge. Mucosal and submucosal herniation through the muscular layer defect increases their propensity to perforate [4].

AD is known to be associated with male gender, advanced age, cystic fibrosis, and Hirschsprung's disease. Appendiceal diverticulitis typically manifests in the fourth and fifth decades and is usually asymptomatic [5]. The clinical manifestations of AD can resemble acute appendicitis, leading to the former diagnosis being overlooked. Leukocytosis, fever, tenderness at the McBurney point, and right iliac fossa pain are all possible signs of both differentials. But appendicitis typically appears before the third decade of life in patients, often accompanied by vomiting, nausea, and anorexia; surgery is often required within a day. On the other hand, AD typically manifests itself after the age of thirty, with an intermittent, insidious onset and subtle pain that starts two weeks prior to the illness [2]. A retrospective study on computed tomography (CT) imaging of patients with acute appendicitis vs AD revealed *peri* appendiceal fat stranding, thickening or enhancement of the appendix, and enlarged appendices in the former group, while the latter demonstrated an absence of fluid level (in the lumen) and appendicolith and the presence of localized abscess formation [6]. Radiological diagnosis of AD can be challenging when there are advanced inflammatory changes or a perforation that may mask the presence of diverticulum [7]. To prevent higher morbidity and mortality due to perforation and peritonitis, early surgical intervention is required.

AD is a rare cause of acute abdomen that is commonly interpreted as acute appendicitis until conclusive histopathology results. As a relatively uncommon condition, AD is typically documented in case reports. Here, we describe cases of two male patients of different ages who underwent appendectomy for symptomatic RIF pain, with a diagnosis of appendicitis. It wasn't until a subsequent pathology report that AD was diagnosed.

This case report has been reported in line with the SCARE criteria [14].

## 2 Case presentation

### 2.1 Case 1

**Fig. 1:**
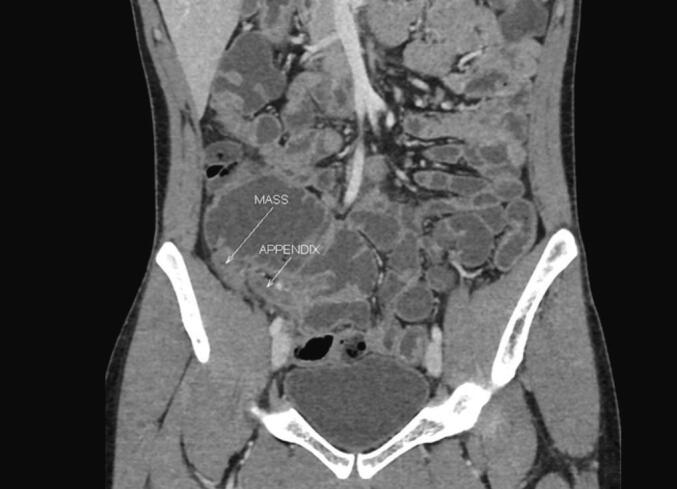
CECT appendicular mass finding in Patient 1.

Patient 1, a twenty-four year-old male, presented to the outpatients department (OPD) with complaints of recurrent right lower abdominal pain and mass (same site) with an onset of one year. He had a past medical history of hypothyroidism (under medical treatment) and gastritis. The episodes of pain were always mild and intermittent, subsiding in 2–3 days. There was no history of vomiting, fever or constipation. He experienced six episodes of pain in the 12 months before presentation, with the last episode occurring 3 months prior to the current presentation and was managed conservatively at the time. Ultrasound abdomen and pelvis showed inflammatory appendiceal mass with features suggestive of chronic contained perforation, a thick-walled collapsed appendix with a tip closely related to mildly thick walled small bowel loops with adjacent mesenteric inflammatory changes and nodes. Follow-up computed tomography scan (CT) showed thick walled appendix lateral to the caecum and terminal ileitis (Fig. 1). Oesophago-gastro-duodenoscopy (OGD) done in the past revealed gastritis, and a colonoscopy to rule out inflammatory bowel disease was negative with a normal terminal ileum biopsy report. The diagnosis, management options, and complications were discussed with the patient, and a plan for elective laparoscopic appendectomy was mutually agreed upon. An endocrinology consultation was sought in view of the high TSH and clearance for surgery obtained.

**Fig. 2:**
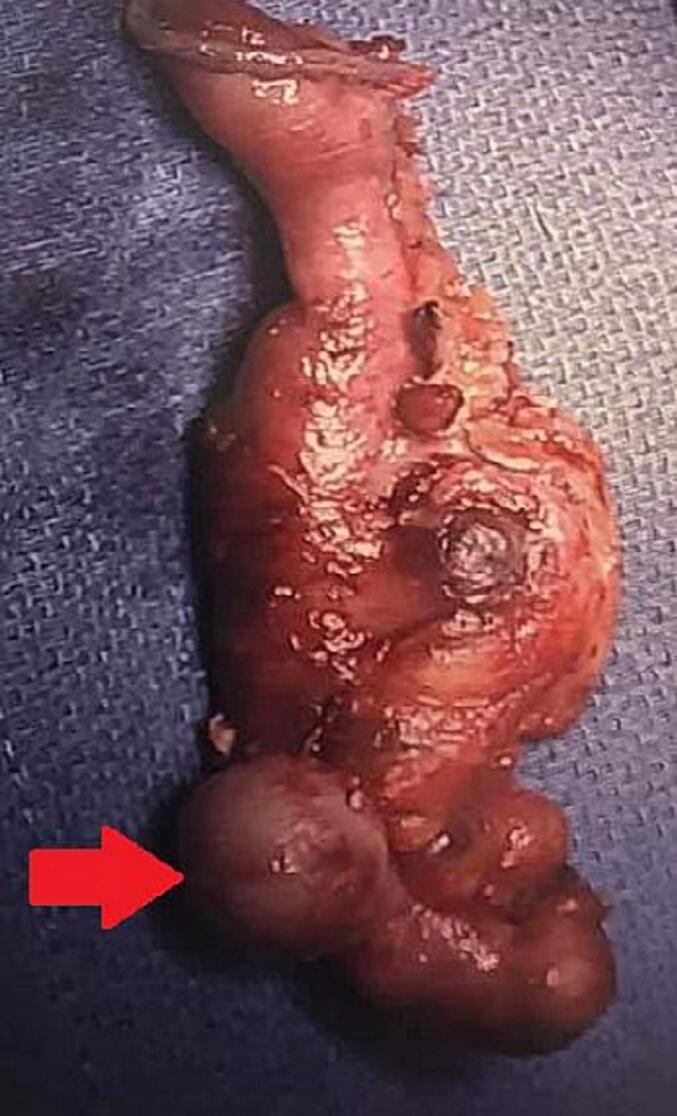
Appendix specimen of Patient 1.

The patient underwent a laparoscopic appendectomy with no complications. Intraoperative findings revealed flimsy omental adhesions to the retrocaecal appendix and post- inflammatory adhesions of the appendix and mesoappendix to the lateral abdominal wall and caecum. There were a few enlarged lymph nodes in the mesoappendix, and a partially mobile caecum, and minimal free fluid in the pelvis (Fig. 2).

Post-operatively, the patient was managed with IV antibiotics, IV fluids, analgesics, and other supportive measures. He was started on oral liquids on the same evening and later changed to a soft diet, which was tolerated well. He recovered symptomatically and was subsequently discharged.

**Fig. 3:**
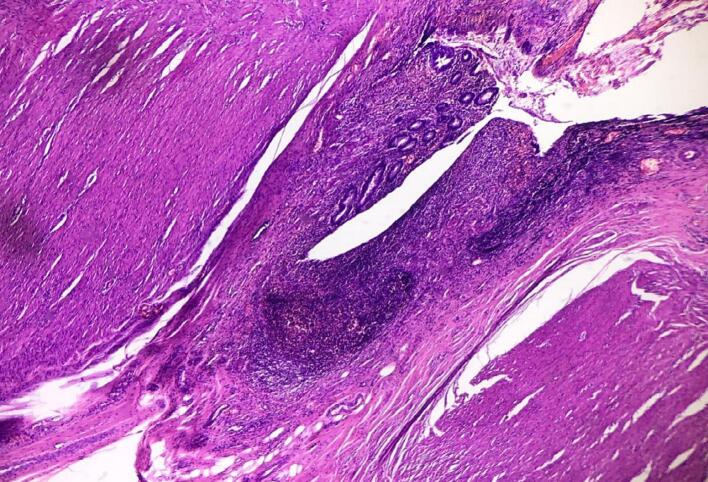
Histopathology image for Patient 1.

Histopathology revealed an appendix specimen with focal mucosal ulceration. Lamina propria and submucosa showed reactive lymphoid follicles. Foci showing herniation of mucosa through the wall of the appendix were noted. Subserosa showed suppurative inflammation, mucinous material, and foreign body giant cell reactions suggestive of healed perforation. A diagnosis of AD with features suggestive of healed perforation was made (Fig. 3).

### 2.2 Case 2

**Fig. 4:**
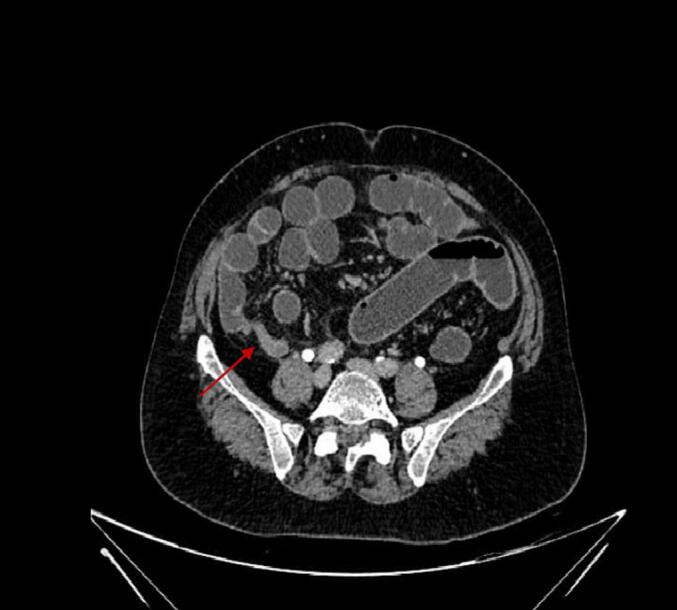
CT appendix body of Patient 2.

Patient 2, a seventy-two-year-old male, was referred to our outpatient clinic with a complaint of worsening right-sided lower abdominal pain for two weeks. There were no other associated symptoms. He had a past medical history of diabetes mellitus (DM) and had recently undergone colonoscopy with polypectomy. CT of the abdomen and pelvis performed after two weeks of onset of symptoms but before presenting to our clinic revealed a thickened appendix measuring 13 mm with adjacent fat stranding. The wall appeared indistinct and irregular near the tip of the appendix with adjacent, mildly ill-defined peri-appendiceal free fluid. These features were suggestive of acute appendicitis with possible sealed-off perforation (Fig. 4). On examination, he had tenderness in the right iliac fossa with no obvious lump or rigidity. After discussion of the clinical status and treatment options with the patient and his relatives, he was admitted for surgical management.

The patient underwent an emergency laparoscopic appendectomy with no complications. Intraoperative findings included a normal base of the appendix, a thickened tip of the appendix, and adhesions to the terminal ileum in the post-ileal position. No lymphadenopathy was noted.

He was discharged on the second postoperative day after an uneventful recovery. Histopathology revealed an appendix specimen showing mucosa lined by tall columnar epithelium with areas of mucosal and submucosal outpouching through muscularis propria. There was a diffuse, dense mixed inflammatory cell infiltrate in the muscularis propria composed of neutrophils, lymphocytes, plasma cells, and scattered eosinophils. No evidence of dysplasia or malignancy was noted. A diagnosis of AD was made.

A comparison of our case characteristics and features is summarized in Table 1.

**Table 1:** Comparison table of characteristics and features of the cases.

Characteristics/symptoms	Patient 1	Patient 2
Age	24 years	72 years
Sex	Male	Male
Comorbidities	Hypothyroidism, Gastritis	DM, colonoscopy with polypectomy
Duration of symptoms	1 year	2 weeks
Migratory pain	No	No
Anorexia	No	No
Nausea	No	No
Tenderness RIF	Yes	No
Rebound tenderness	No	No
Elevated temperature	No	No
Leukocytosis	No	No
Imaging findings	Inflammatory appendicular mass with chronic contained perforation	Thickened appendix with adjacent fat stranding and peri-appendiceal fluid
Intra-op findings	Post inflammatory adhesions of appendix and mesoappendix to lateral abdominal wall and caecum	Thickened tip of appendix, adherent to terminal ileum, in the post-ileal position
Histopathology findings	Herniation of mucosa through the wall of appendix with suppurative inflammation suggestive of healed perforation	Areas of mucosal and submucosal outpouchings through muscularis propria with diffuse dense mixed inflammatory cell infiltrate in the muscularis propria

## 3 Discussion

Appendiceal diverticulitis is a rare but significant form of appendiceal disease that can mimic acute appendicitis. It is crucial to comprehend the aetiology and clinical progression of AD, whether it develops gradually or is found by chance during an appendectomy, in order to effectively manage it. Although the aetiology is unknown, males above the age of 30 are usually affected, as described in the report of 61 cases of AD by Deschenes et al. [8]. Albaugh et al., on the other hand, noted that the incidence of AD in patients under 30 having appendectomy is less than 1 % [9]. Our cases report two men who fall into both age categories. AD is a distinct disease process that presents differently from classic appendicitis in terms of age, symptom onset, surgical findings, and histopathological analysis. A complete medical history and physical examination may occasionally be used to differentiate between these two conditions. Some patients may disclose past episodes of lower quadrant pain on the right side, keeping in mind chronic appendicitis. The same trend can be seen in both our patients, where the onset of symptoms was indolent over weeks or months and involved repeated occurrences. Appendicitis can present as localized or generalized peritonitis, while in AD, the inflammatory process is kept within the mesoappendix by surrounding adhesions due to chronic insults. It is common to mistakenly diagnose this “mass” effect as carcinoma [2]. Our first patient had a mass in the area of clinical concern with lower abdominal pain. US findings were consistent with an inflammatory appendiceal mass with characteristics that suggested a chronic perforation.

Although reports of preoperative diagnosis of AD on CT scan or ultrasound have been published [10], both our cases were diagnosed as acute appendicitis. AD occurring in the absence of classic imaging findings and luminal obstruction presents a diagnostic dilemma and needs further research.

Table 1 shows a comparison between the two cases in this report, and it can be inferred that the most commonly used clinical scoring system for diagnosing acute appendicitis, called the Alvarado score, could not be used as a clinical prediction score for AD. The scale, also referred to by its acronym, MANTRELS, consists of two laboratory measurements and six clinical items (three signs and three symptoms). Each item is assigned an additive point score, with a maximum of 10 points achievable [11]. While our report's radiology imaging concluded acute appendicitis for both patients, the clinical scoring system only recorded a positive result for RIF tenderness in the first case. This mandates additional study on AD and the creation of comparable scoring systems in the future to aid in diagnosis.

Based on Lipton et al.'s classification of appendiceal diverticular diseases, our case fits the criteria for microscopic type 1, primary acute diverticulitis, and acute peri-diverticulitis [12] (Table 2). While colonic diverticulitis (Hinchey 1: diverticulitis with pericolic abscess, 2: diverticulitis with distant abscess) is treated conservatively, similar AD appears to persist even after antibiotics and require surgery. Accurate diagnosis and surgical intervention are crucial due to the risk of perforation (more than four times as likely as acute appendicitis) [12]. AD is also associated with a risk of carcinoid and mucinous malignancies, peritoneal seeding, and pseudomyxoma peritonei. A retrospective study by Lamps et al. demonstrated there is a statistically significant (*P* < .001) correlation between diverticula and low-grade mucinous neoplasms in the appendix specimens post-appendectomy [13]. The same study also concluded that the presence of a low-grade tumour inside the diverticulum may cause the distending mucin to rupture and produce pseudomyxoma peritonei. The biopsy showed no signs of cancer in either patient in our report. The pathologic mechanism leading to perforation is also not understood, since there is minimal peristalsis in the appendix and neither of our patients had any luminal obstruction, which could explain the perforation of the pseudodiverticula.

**Table 2:** Appendicular diverticulitis classification by Philip et. al [12].

Type	Classification
Type 1	Primary Acute diverticulitis, with or without peri-diverticulitis
Type 2	Acute diverticulitis secondary to acute appendicitis
Type 3	Diverticulum without inflammation
Type 4	Diverticulum with acute appendicitis
Type 5	Chronic peri-diverticulitis with acute appendicitis

## 4 Conclusion

Although AD and acute appendicitis share similarities, AD is a separate condition that should be suspected in patients presenting with RIF pain, particularly if they are older and have a longer duration of symptoms. The natural history and complications of AD seem to be different from the classical cases of acute appendicitis. AD is likely to be associated with higher rates of perforation peritonitis and mucinous neoplasms. Conservative management of AD may not be beneficial, unlike conservative management of selected cases of acute appendicitis or colonic diverticulitis; however there are no guidelines or meta-analyses to support this. Further research is needed to aid diagnosis, stratify the risk and develop guidelines for the management of AD.

## Consent

Written informed consent was obtained from the patient for publication of this case report and accompanying images. A copy of the written consent is available for review by the Editor-in-Chief of this journal on request.

## Ethical approval

All ethical considerations have been taken into account. No personal data has been exposed.

## Funding

This research did not receive any specific grants from funding agencies in the public, commercial, or not-for-profit sectors.

## Author contribution

Writing, Literature search, Methodology and Editing: Dr. Midhun Mathew.

Data curation, Editing and Analysis: Dr. Mohammed Fajar Al Sadiq.

Conceptualization, Reviewing and Editing: Dr. Vinod Gopalkrishna Pillai.

## Guarantor

Midhun Mathew, Mohammed Fajar Al Sadiq, Vinod Gopalkrishna Pillai.

## Research registration number

Our study is not a first in man case report.

## Conflict of interest statement

None declared.
